# Home Oral Care Domiciliary Protocol for the Management of Dental Erosion in Rugby Players: A Randomized Clinical Trial

**DOI:** 10.3390/jcm11164893

**Published:** 2022-08-20

**Authors:** Andrea Butera, Simone Gallo, Maurizio Pascadopoli, Giuseppe Alessandro Scardina, Sofia Pezzullo, Andrea Scribante

**Affiliations:** 1 Unit of Dental Hygiene, Section of Dentistry, Department of Clinical, Surgical, Diagnostic and Pediatric Sciences, University of Pavia, 27100 Pavia, Italy; 2 Unit of Orthodontics and Pediatric Dentistry, Section of Dentistry, Department of Clinical, Surgical, Diagnostic and Pediatric Sciences, University of Pavia, 27100 Pavia, Italy; 3 Department of Surgical Oncological and Stomatological Disciplines, University of Palermo, 90127 Palermo, Italy

**Keywords:** dental erosion, demineralization, erosion, white spot lesions, physical activity, sport drinks, rugby players, athletes, hydroxyapatite, remineralization

## Abstract

People performing regular physical activity are at high risk of dental erosion especially in cases of high sport drinks intake. Biomimetic hydroxyapatite-based oral hygiene products, like toothpastes and mouthwashes, have been investigated in recent years for their remineralizing activity on the teeth. The aim of the present study was to evaluate the efficacy of two different oral hygiene protocols, respectively consisting of the combination of a hydroxyapatite-based toothpaste plus mouthwash (Trial group) or toothpaste alone (Control Group). At baseline (T0), as well as at 15 days (T1), 30 days (T2) and 90 days (T3), the following clinical indexes were assessed: Basic Erosive Wear Examination (BEWE), Schiff Air Index (SAI), Visual Analogue Scale (VAS), Plaque Index (PI) and Bleeding Index (BI). In general, for all the indexes assessed, a progressive intragroup reduction was noticed from the baseline to the subsequent timepoints, with no intergroup differences. Accordingly, the use of the hydroxyapatite-based toothpaste, alone or in combination with the mouthwash containing hydroxyapatite as well, is an effective method for the domiciliary management of dental erosion in physically active individuals like rugby players.

## 1. Introduction

Enamel demineralization is one of the major concerns in dentistry and several studies have been conducted on this topic, both in children and in adults [1,2]. The arise of white spot lesions (WSLs) on the tooth surface is the first sign of dental caries. This alteration of the enamel structure, besides being inaesthetic, might also be responsible of dental hypersensitivity [3]. Considering its supersaturation with calcium (Ca^2+^) and phosphate (PO_4_^3−^) ions, saliva has a protective role towards enamel erosion with these ions being able to diffuse into deficient lesions, thus promoting remineralization [4]. However, this process is insufficient to completely repair the enamel, therefore additional remineralizing agents are required [5]. Until now, fluoride has been extensively used for both caries prevention and the remineralization of early lesions, however some concerns are related to its use [6,7]. For instance, its efficacy is decreased when the oral cavity pH is under 4.5, moreover, an adequate concentration of Ca^2+^ and PO_4_^3−^ is necessary for fluoride to exert its action [4]. Additionally, only the outer layer of the enamel takes advantage of this remineralizing process, whereas the core of the lesion is not altered [5]. Finally, another shortcoming associated with fluoride use is the risk of fluorosis and toxicity [8]. On the basis of these considerations, new agents have been proposed, and one of the most recent technologies is represented by the use of biomimetic hydroxyapatite (HAP) in form of a microcluster or in nanocrystalline form [9,10]. This substance is chemically similar to the apatite constituting the human enamel crystals, and previous research has demonstrated the deposition of biomimetic hydroxyapatite on the tooth [11]. 

Dental erosion is a form of tooth wear that can be found both in deciduous and permanent dentition; its prevalence is increasing in the recent years, and this involves a variegated etiology: dietary habits, lifestyle, drugs, exposure to acidic substances, and elevated gastric acidity due to comorbidities. Regular visits from the dentist can help in preventing this condition. The treatment should consider including not only dental specialists, but also medicine specialists, given the fact that it is a multifactorial issue [12]. Almost a half of the professional players were found to suffer from dental erosion, with a significant correlation with sports drink use [13]. In detail, rugby players exhibit poor oral conditions with a higher prevalence of DMF scores, gingivitis and dysbiotic oral microbiota, with a prevalence of *Streptococcus* [14]. Current guidelines agree in adopting a multidisciplinary approach in the management of dental erosion in those kind of patients [15]. 

To date, the impact of sport training on the oral health has been already addressed in literature [16]. However, due to the lack of studies investigating the approaches of management for dental erosion in athletes, the aim of the present study was to compare the efficacy of two different domiciliary oral hygiene protocols based on biomimetic hydroxyapatite in physically active people like rugby players. The null hypothesis of the study was that no significant differences occurred between the two different protocols tested.

## 2. Materials and Methods

### 2.1. Trial Design

This was a single-center, parallel-group, randomized clinical trial with a 1:1 allocation ratio, approved by the Unit Internal Review Board (registration number: 2021-0908) and registered on Clinicaltrials.gov (NCT number: NCT05140538).

### 2.2. Participants

Patients registered for periodontal care at the Unit of Dental Hygiene, Section of Dentistry, Department of Clinical, Surgical, Diagnostic and Pediatric Sciences of the University of Pavia (Pavia, Italy) were enrolled for the study. The aim of the study was explained to patients that fulfilled the inclusion criteria and asked to participate. After signing the informed consent, they were enrolled from December 2021 to January 2022. The study ended in May 2021. Both interventions and outcomes assessment were conducted at the same Unit. 

The inclusion criteria were being aged between 18 and 70 years; being a rugby player that used a mouthguard; being a patient willing to participate to the study. The following were the exclusion criteria: patients with a cardiac pacemaker; patients suffering from neurological disorders; patients suffering from psychological disorders; pregnant or breastfeeding women.

### 2.3. Interventions and Outcomes

At the first appointment (T0), patients were asked to sign the informed consent document to participate to the study. An instructed operator collected the following periodontal clinical indexes by means of a probe (UNC probe 15; Hu-Friedy, Chicago, IL, USA): Plaque Index (PI) (values from zero to three, respectively representing no plaque, thin plaque layer, moderate plaque layer, and abundant plaque layer) [17]; Bleeding on Probing (BOP) (values from zero to three, respectively representing no inflammation with no bleeding, mild inflammation with no bleeding, moderate inflammation with bleeding on probing, and severe inflammation with tendency to spontaneous bleeding) [11]; BEWE index assessed with Intact Tooth^®^ smartphone application (values from zero to three, respectively representing no erosion, initial loss of surface texture, hard tissue loss less than 50%, and hard tissue loss more than 50%) [17]; Visual Analogue Scale (VAS) for dental sensitivity (values from zero to 10 referred by the patient) [18]; Schiff Air Index (values from zero to three, respectively representing no response to air stimulus, response to air stimulus but with no request of discontinuation, response to air stimulus and request of air discontinuation, and response to air stimulus perceived as painful and request of discontinuation) [18]. Then, a professional supragingival and subgingival oral hygiene procedure was conducted using a piezoelectric instrument (Multipiezo, Mectron S.p.a, Carasco, Italy) and Gracey curettes (Hu-Friedy, Chicago, IL, USA).

Participants received verbal instructions on domiciliary oral hygiene with a soft-bristled electric toothbrush to be used twice a day for 2 min. At this stage, they were divided into two groups according to the assigned home treatment: in the Trial group, Biorepair Total Protective Repair toothpaste and Biorepair Triple Action Mouthwash were used twice a day; in the Control group, only Biorepair Total Protective Repair toothpaste was used twice a day. The planned follow-ups were conducted after 15 days (T1), 30 days (T2), and 90 days (T3). At each appointment, the periodontal examination with the collection of clinical indexes was performed. 

Participants were recommended to regularly and correctly clean their teeth [19]. 

The composition of the products that were tested is shown in Table 1. 

### 2.4. Sample Size

Sample size calculation (Alpha = 0.05; Power = 80%) for two independent study groups and a continuous primary endpoint was calculated considering the “BEWE index” variable. The expected mean was supposed to be 1.07 with a standard deviation of 1.82, and the expected difference between the means was supposed to be 1.15, therefore 20 patients per group were required for the study [20]. 

### 2.5. Randomization and Blinding

By means of a block randomization table, the data analyst provided a randomization sequence, considering a permuted block of 40 total participants. An operator enrolled the participants and executed the professional oral procedures, collecting all the above-mentioned indexes. On the basis of previously prepared sequentially numbered, opaque, sealed envelopes (SNOSE), an assistant assigned each participant to the respective group, concealing the products for home use. The data analyst was blinded for the allocation and outcomes. 

### 2.6. Statistical Methods

Data were submitted for statistical analysis using R Software (R version 3.1.3, R Development Core Team, R Foundation for Statistical Computing, Wien, Austria). For each group and variable, descriptive statistics (mean, standard deviation, minimum, median, and maximum) were calculated. BEWE, SAI, and VAS were calculated as pure values; BOP and PI were calculated in percentages. Data normality was assessed using the Kolmogorov–Smirnov test. For each variable, an ANOVA test was performed, detecting significant differences among the groups. Lasty, inferential comparisons using a post hoc Tukey test were performed.

Significance was predetermined at *p* < 0.05 for all the tests performed. 

## 3. Results

### 3.1. Participant Flow and Baseline Data

**Figure 1:**
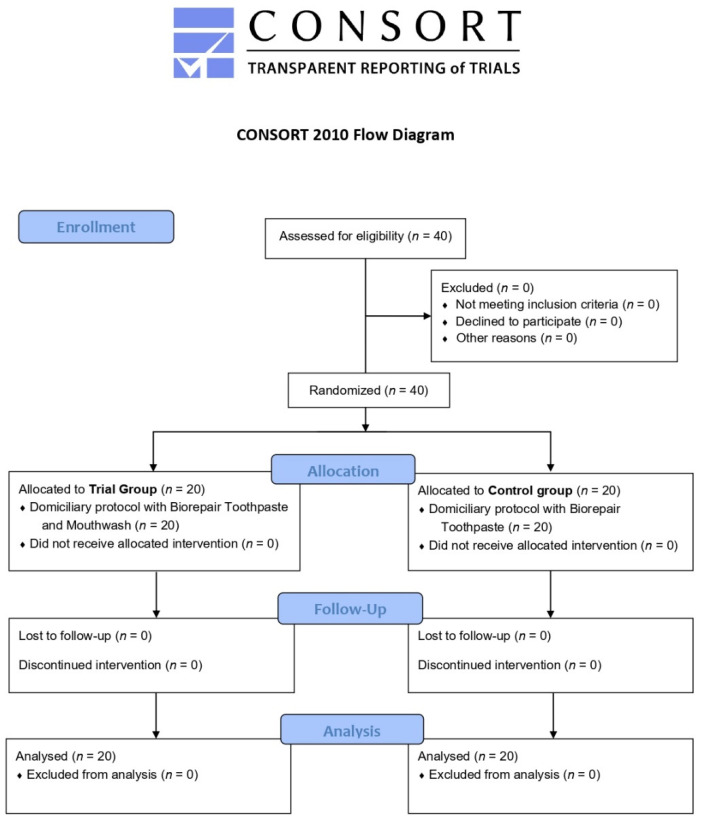
CONSORT flow chart of the study.

A total of 40 patients responded to the inclusion criteria and they were asked to participate in the study. They all agreed to participate and received the allocated interventions. No patient was excluded from the analysis. The flow chart of the study is shown in Figure 1. The study sample consisted of male patients, showing a mean age of 26.28 ± 5.55 years (20 patients for the Trial group, with a mean age of 26.9 ± 6.01; 20 patients for the Control group, with a mean age of 25.65 ± 5.12).

Descriptive and inferential statistics for the five variables tested are reported in the following sections. Intergroup and intragroup comparisons are shown using a documented letter-based comparison system so that the presence of the same letter/letters for compared means shows that no significant differences are present between them [21]. Additionally, the letter-based significance description has been widely used in previous recent reports [22,23,24].

### 3.2. Basic Erosive Wear Examination (BEWE)

**Figure 2:**
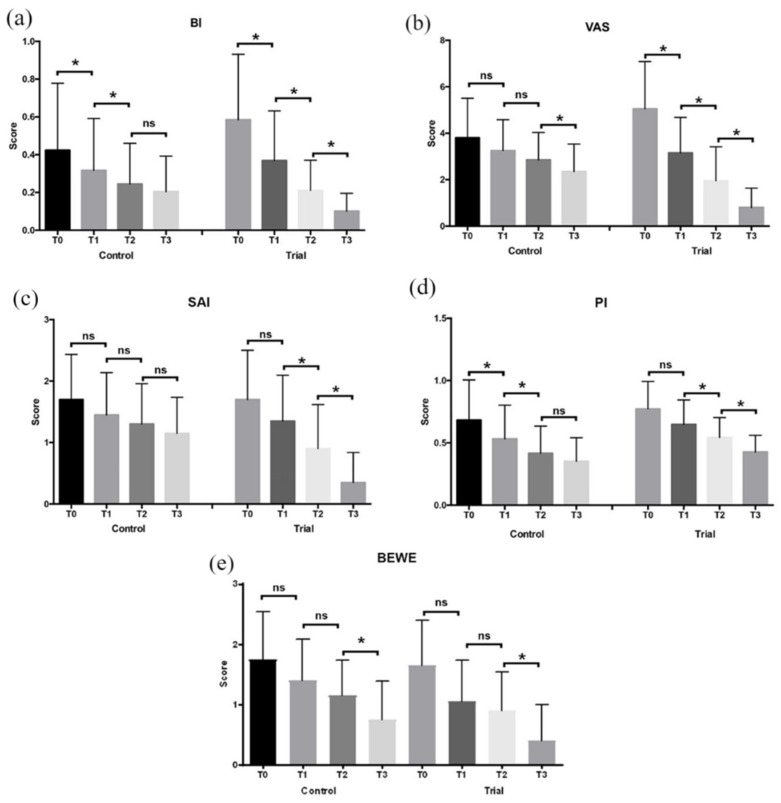
Diagrams of the clinical indexes (mean and SD) assessed at T0, T1, T2, and T3: (**a**) Bleeding Index; (**b**) Visual Analogue Scale; (**c**) Schiff Air Index; (**d**) Plaque Index; (**e**) Basic Erosive Wear Examination. Legend: ns: not significant; *: *p* < 0.05.

The results of the BEWE examination are shown in Table 2 and Figure 2. For both the groups, a decrease of the BEWE scores was assessed along all the time frames of the study. No significant intergroup comparisons were found (*p* > 0.05). A significant decrease was found between T2 and T3 time frames for both of the groups (*p* < 0.05). 

### 3.3. Schiff Air Index (SAI)

The results of the SAI are shown in Table 3 and Figure 2. A reduction of the tested index was assessed along the time frames of the study. A significant intragroup reduction can be found from T1 to T2 for both the groups, and from T2 to T3 only for Trial Group (*p* < 0.05). No intergroup differences can be found among the groups except that during T3, the Trial Group significantly differs from all the time frames (*p* < 0.05). 

### 3.4. Visual Analogue Scale for Dental Sensitivity (VAS)

The results of the VAS are shown in Table 4 and Figure 2. The VAS scores decreased in both the groups during the study. Intragroup-significant reduction can be found only between T2 and T3 for the Control Group, and between all the time frames of the Trial Group (*p* < 0.05). In regards for the intergroup differences, a significant difference can be found between the two groups only for the T3 time frame (*p* < 0.05).

### 3.5. Plaque Index (PI%)

The results of the PI are shown in Table 5 and Figure 2. A reduction of the PI scores was assessed in both the groups. Lower values can be found in the Control Group for all the time frames of the study, however no significant intergroup and intragroup difference was found (*p* > 0.05). 

### 3.6. Bleeding Index (BI%)

The results of the BI index are shown in Table 6 and Figure 2. The BI mean values significantly decreased between T0 and T1 in both the groups (*p* < 0.05). No significant intergroup differences were found at each time frame (*p* > 0.05). 

## 4. Discussion

Dental erosion consists of the loss of the enamel and the dentine structure, which are chemically dissolved by exogenous or endogenous acids. One of the categories that is most affected by tooth erosion is represented by people undergoing regular physical activity with a high consumption of sports drinks [25,26]. Such beverages mainly contain citric acid, an organic acid which causes a reduction of the salivary pH with dissolution of the calcium ions constituting the enamel [27,28]. Moreover, other factors related to the specific sport could have an influence; for instance, water sports professionals are extremely exposed to erosive lesions due to the exposition to chlorinated water [29].

In their recent systematic review with meta-analysis that is aimed at establishing a relationship between dental erosion and regular physical activity with or without sports drink consumption, Nijakowski and colleagues [25] included 16 studies reporting the prevalence of erosion in physically active individuals and thus determined that the aggregate prevalence was approximately 46.55% [95% CI: 36.10–57.15%]. Similarly, in this group, the summarized frequency of the consumption of sports drinks was estimated to be around 56.02% [95% CI: 29.70–80.64%]. Altogether, physically active individuals who declare regular consumption of sports drinks have a more than 2.5-fold increase in the odds of developing erosive lesions; in general, almost half of the people who practice sports suffer from tooth erosion, and more than half frequently consume sports beverages. Upon the limitations of their review, like the heterogeneity of the included studies encompassing different age groups and various sport disciplines, the authors concluded that regular physical activity was associated with an increased risk of dental erosion, especially under the influence of the frequent consumption of sports drinks.

The aim of the present study was to compare the efficacy of two different domiciliary oral hygiene protocols based on biomimetic hydroxyapatite use in the management of dental erosion in rugby players. The null hypothesis was partially rejected considering that significant differences were found. For both the groups, respectively assigned to the combination of hydroxyapatite-based toothpaste plus mouthwash and toothpaste alone, a significant decrease in the BEWE scores was found between T2 and T3, whereas no significant intergroup comparisons were found. Considering the Schiff Air Index (SAI), a significant intragroup reduction was found between T1 and T2 for both the groups and between T2 and T3 this was only for the Trial Group; conversely, no intergroup differences were found among the groups except for T3 of the Trial Group. In regards to the VAS scores, they decreased in both the groups during the study—an intragroup-significant reduction was found only between T2 and T3 for the Control Group and between all the time frames of the Trial Group. Additionally, a significant intergroup difference was found between the two groups only for the T3 time frame.

In addition to the abovementioned parameters, even indexes related to oral hygiene, like Plaque Index (PI) and Bleeding Index (BI) were assessed. A reduction in the PI scores was assessed in both the groups. Lower values were found in the Control Group for all the time frames of the study, however no significant intergroup and intragroup differences were found. Finally, the BI mean values significantly decreased between T0 and T1 in both the groups and no significant intergroup differences were found at any time frame. 

On the basis of the abovementioned results, both the combination of the hydroxyapatite-based toothpaste plus mouthwash and the toothpaste alone was generally effective in improving enamel erosion and oral hygiene parameters. To the best of our knowledge, this is the first study evaluating the action of biomimetic hydroxyapatite for the dental erosion of rugby players, therefore no direct comparisons of the results obtained can be done. 

Previous studies have demonstrated the deposition of biomimetic hydroxyapatite contained in products for oral care. In particular, in a previous work by our group, the deposition of calcium, phosphorus, and silicon ions on the surfaces of bulk-filled polymeric composite resins in the oral environment after one month of daily oral hygiene with a toothpaste containing microRepair^®^ (Zn-carbonate hydroxyapatite) was demonstrated by means of SEM and EDS analyses [30]. In the study by Lelli and colleagues [31], two groups of patients between 18 and 75 years old were assigned to the use of a Zn-CHA nanocrystals-based toothpaste (experimental group) and a potassium nitrate/sodium fluoride toothpaste (active control group) for 8 weeks. At the end of this period, extractions were performed in five subjects per study group. Negative controls were represented by two subjects treated with non-specified fluoride toothpaste. Teeth were then analyzed by means of Scanning Electronic Microscopy and with Elementary analysis, X-Ray Diffraction analysis, and Infrared analysis. The results showed that the use of the Zn-CHA nanocrystals toothpaste led to a remineralization/repair of the enamel surface, by deposition of a hydroxyapatite-rich coating. Conversely, the use of both nitrate potassium/sodium fluoride and non-specified fluoride toothpastes did not appreciably alter the enamel surface. Despite our study being based on a different methodology, the results obtained by our group confirm those that are mentioned above. 

The main limitation of the present work is represented by the assessment of only clinical indexes with no morphological and chemical analyses on extracted teeth. Moreover, no negative controls consisting of other chemical compounds (like fluoride) have been considered. Future studies with longer follow-ups and those taking into account further remineralizing agents are thus required to confirm the results here obtained. In particular, SEM and EDS analyses are necessary to demonstrate the real deposition of hydroxyapatite crystals on the tooth surfaces in order to strengthen the findings here that were obtained clinically. 

## 5. Conclusions

People practicing sport, like rugby players, are exposed to the risk of tooth erosion due to the sport drinks intake. The findings of the present study show that the combination of a hydroxyapatite-based toothpaste plus mouthwash or toothpaste alone could be effective in remineralizing enamel, besides improving oral hygiene indexes. 

## Figures and Tables

**Table 1:** Materials used in the study and their compositions.

Product	Manufacturer	Composition
Biorepair Total Protective Repair	Coswell S.p.A., Funo di Argelato, BO, Italy	Aqua, Zinc Hydroxyapatite (microRepair^®^), Glycerin, SorbitoI, Hydrated Silica, Silica, Aroma, Cellulose Gum, Tetrapotassium Pyrophosphate, Sodium Myristoyl Sarcosinate, Sodium Methyl Cocoyl Taurate, Sodium Saccharin, Citric Acid, Phenoxyethanol, Benzyl Alcohol, Sodium Benzoate.
Biorepair Triple Action Mouthwash	Coswell S.p.A., Funo di Argelato, BO, Italy	Aqua, Sorbitol, Xylitol, Zinc PCA, Cellulose Gum, Zinc Hydroxyapatite (microRepair^®^), Aroma, Sodium Lauryl Sulfate, Mentha Arvensis Leaf Oil, PEG-40 Hydrogenated Castor Oil, Sodium Myristoyl Sarcosinate, Sodium Methyl Cocoyl Taurate, Sodium Saccharin, Sodium Benzoate, Benzyl Alcohol, Phenoxyethanol, Limonene.

**Table 2:** Descriptive statistics of BEWE measurements.

Group	Time	Mean	Standard Deviation	Significance *
Control Group	T0	1.75	0.79	A
	T1	1.40	0.68	A, B
	T2	1.15	0.59	B
	T3	0.75	0.64	C, D
Trial Group	T0	1.65	0.75	A
	T1	1.05	0.69	A, B, C
	T2	0.90	0.64	A, B, C
	T3	0.40	0.60	D

* Means with same letters do no show statistically significant differences (*p* > 0.05).

**Table 3:** Descriptive statistics of SAI measurements.

Group	Time	Mean	Standard Deviation	Significance *
Control Group	T0	1.70	0.73	A, B
	T1	1.45	0.69	A, B, C, D
	T2	1.30	0.66	D, E
	T3	1.15	0.59	D, E
Trial Group	T0	1.70	0.80	A, C, D
	T1	1.35	0.75	A, C, D
	T2	0.90	0.72	B, E
	T3	0.35	0.49	F

* Means with same letters do no show statistically significant differences (*p* > 0.05).

**Table 4:** Descriptive statistics of VAS measurements.

Group	Time	Mean	Standard Deviation	Significance *
Control Group	T0	3.80	1.70	A, B, C
	T1	3.25	1.33	C, D, E
	T2	2.85	1.18	E, F
	T3	2.35	1.18	G, H
Trial Group	T0	5.05	2.04	A
	T1	3.15	1.53	C, F, G
	T2	1.95	1.47	E, H
	T3	0.80	0.83	I

* Means with same letters do no show statistically significant differences (*p* > 0.05).

**Table 5:** Descriptive statistics of PI% measurements.

Group	Time	Mean	Standard Deviation	Significance *
Control Group	T0	68.00	32.00	A, B, C
	T1	53.00	27.00	D, E, F
	T2	42.00	22.00	G, H
	T3	35.00	19.00	G, I
Trial Group	T0	77.00	22.00	A
	T1	65.00	20.00	A, D
	T2	54.00	16.00	B, E, H
	T3	43.00	14.00	F, G

* Means with same letters do no show statistically significant differences (*p* > 0.05).

**Table 6:** Descriptive statistics of BI% measurements.

Group	Time	Mean	Standard Deviation	Significance *
Control Group	T0	42.00	35.00	A, B, C
	T1	32.00	28.00	D, E, F
	T2	24.00	22.00	G, H, I
	T3	20.00	19.00	G, H, I
Trial Group	T0	58.00	35.00	A, D
	T1	37.00	26.00	B, E, H
	T2	21.00	16.00	C, F, G
	T3	10.00	09.00	I

* Means with same letters do no show statistically significant differences (*p* > 0.05).

## Data Availability

All data are available upon request to corresponding authors.
